# 

**Published:** 1890-09

**Authors:** 


					﻿SEPTEMBER, 1890-
OLD SERIES
VoL XXV
(7 1855 4
NEW SF-RtKS
Vol. VII.-No. 7.

nURGW
rfuURNA
H. V. M. MILLER. M.D., LL.D.
VIRGIL 0. HARDON, M.D.
F. W. MeRAE. M.D..	Ed.
JAMES P. HARRISON 8tC
SUBSCRIPTION! $2.60 A YEAR
				

